# Comparison between menstrual cups: first step to categorization and improved safety

**DOI:** 10.1177/17455065211058553

**Published:** 2021-11-19

**Authors:** Hannah Manley, John A Hunt, Lívia Santos, Philip Breedon

**Affiliations:** 1 Medical Engineering Design Research Group, Nottingham Trent University, Nottingham, UK; 2 Medical Technologies Innovation Facility, Nottingham Trent University, Nottingham, UK; 3 College of Biomedical Engineering, China Medical University, Taichung, Taiwan; 4 Sport, Health and Performance Enhancement (SHAPE) Research Centre, Nottingham Trent University, Nottingham, UK

**Keywords:** comparison, medical device, menstrual cup, menstrual hygiene product, menstrual management, menstruation, women’s health

## Abstract

**Objectives:**

Menstrual cups come in a range of shapes, sizes, and firmnesses, but unlike tampons are not categorized in any way. With these factors having an impact on product leaks and comfort, as well as being linked to illness and injury, women need the same level of transparency when purchasing a menstrual cup. The comparison of physical and mechanical properties of menstrual cups will be the first step to achieve this.

**Methods:**

In October 2020, 14 popular and highly rated menstrual cups underwent quantitative comparison in laboratory settings (the United Kingdom), and they were compared in terms of their dimensions, volume, and compressive strength (firmness) using the Instron Universal Testing System. The overall designs were compared including shape, material, and features.

**Results:**

Although all the products in this comparison were marketed to women below 30 years of age having never given birth, total volume varied from 18.88 mL to 38.14 mL, and compressive load to compress the menstrual cup 50% (±0.5%) maximum diameter varied from 3.39 N to 13.92 N.

**Conclusions:**

Women are not sufficiently informed when choosing a menstrual cup. With no correlation between menstrual cup size, shape, and its volume, or material, shape, and its firmness, consumers cannot estimate which menstrual cup might be most suitable, and incorrect choice could cause injury. Transparency is needed across menstrual cup brands. With this and further regulation, women will make an informed decision to choose the correct menstrual cup and minimize injury. This work recommends firmness categories, ranging from ‘very soft’ to ‘very firm’ as a first step.

## Introduction

Menstrual cups are vessels made of silicone, thermoplastic elastomers (TPE), or natural rubber, designed to be worn vaginally to collect menstrual blood for up to 12 h before being emptied, cleaned, and reinserted. Market analysis shows 4% women in the United Kingdom (UK) use menstrual cups as their preferred menstrual hygiene product of choice.^1^ They are available in a variety of shapes, with additional features available such as valves and anti-spill lips. A systematic review by Van Eijk et al.^2^ identified that by 2019, there were 199 brands of menstrual cups on the market, available in 99 countries, with 145 of these brands ranging in price from US$0.72 to US$46.72. No scientific, quantitative, peer-reviewed menstrual cup comparison exists between more than two menstrual cups, and literature often focuses on women’s attitudes to menstrual cups rather than their physical and mechanical properties.

With menstrual cups lasting up to 10 years,^2^ women report choosing a menstrual cup for economic,^3–5^ comfort,^6,7^ and environmental reasons.^5,8,9^ Generally, women prefer the menstrual cup to previously used methods of menstrual hygiene management (pads or tampons),^10^ and menstrual cup satisfaction has been reported across multiple locations, (e.g. India,^11^ Iran,^12^ North America,^13^ South Africa,^6,14^ Thailand,^15^ and the UK,^5^ with many other studies not reporting location).^2^ Studies have reported that menstrual cup users require familiarization to the product; one study reported that 23% of women had difficulties using the menstrual cup during first cycle use, citing difficult insertion and discomfort. However, once familiarized, more than 90% of participants found the menstrual cups easy to use by their third cycle of use and would recommend to others.^16^

User reviews have been published surrounding menstrual cup use. Shihata and Brody^17^ asked 834 participants to compare the Femmycycle menstrual cup against the menstrual cup they were already comfortable using, but it is unclear whether participants used the product, or reviews were based on seeing it. Online user reviews have largely depended on visual inspection and ‘squeeze tests’ of one or two products.^18^ In online menstrual cup comparison charts, firmness is rated out of five,^19,20^ although categorization methods are not reported. Where user reviews have their value, standardized details on menstrual cup dimension, shape, material, and firmness would inform consumers, and provide context to the user reviews.

Here, it is argued that women are not sufficiently informed when using a menstrual cup for the first time, leading to women choosing a menstrual cup that is not suitable. With this having safety and comfort implications, this article provides a comparison and menstrual cup categorization to allow women to make educated decisions when choosing a menstrual cup.

### Function and safety

**Figure 1.**
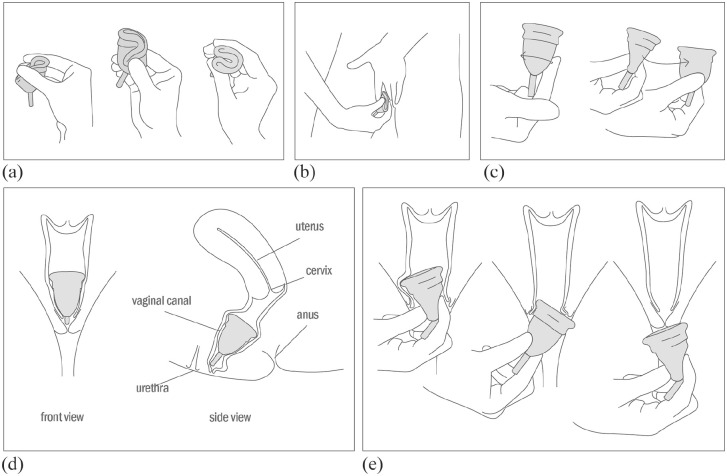
Menstrual cup use. (a) Users find a fold suitable for them; punchdown-fold, 7-fold, and c-fold illustrated. (b) The folded menstrual cup is inserted and allowed to spring open. (c) To ensure the menstrual cup is fully open and therefore creating a seal against the vaginal wall, users can run a finger around the vessel to feel for bumps as a sign it is not open, or users can gently pinch and twist the menstrual cup. (d) The menstrual cup sits lower than a tampon. The stem may need to be trimmed to avoid discomfort. The length of the vaginal canal will affect where the menstrual cup sits in relation to the cervix. (e) To remove, users must ensure the seal is broken by running a finger cup the side of the menstrual cup, or pinching the base of the vessel. The cup is then gently removed, and it can help shift the menstrual cup from side to side.

Menstrual cups are folded before insertion. The menstrual cup is then allowed to spring open, forming a seal against the vaginal wall. For removal, the base of the cup is pinched, and the product moved gently side to side while pulling down (see Figure 1).

Issues arise with incorrect sizing or firmness, see Table 1. If the menstrual cup is too small or the menstrual cup is too soft and the pressure from the vaginal wall prevents the menstrual cup from unfolding, it will leak. A rim with a smaller diameter may become suctioned around the cervix. If the menstrual cup is too large or firm, it is uncomfortable for the wearer and could cause injury.

**Table 1.** Menstrual cup improper fit matrix.

	Potential minor issues	Potential major issues
Too small	Menstrual cup will not form a seal against the vaginal wall, causing leaks.	Menstrual cup rim could suction around the cervix, causing pain or prolapse if pulled during removal.
Too soft	Menstrual cup will not open, causing leaks.	–
Too large	Discomfort experienced during insertion and removal.	Obstruction of urine flow, causing renal colic. Difficulty removing menstrual cup could cause prolapse.
Too firm	Discomfort experienced during insertion and removal.	Obstruction of urine flow, causing renal colic.

The majority of evidence shows that menstrual cups are safe to use.^2^ Rare cases of renal colic have been reported, caused by a menstrual cup blocking the urinary tract.^21–23^ These could be attributed to menstrual cups being too large or firm and exerting pressure on the tissues surrounding the vagina. Similarly, a case of hydroureteronephrosis (blockage leading to kidney breakdown) was caused by ‘deeply inserted’ menstrual cup suctioning on the fornix (the recess from the protrusion of cervix).^24^ This could be caused by a smaller rim diameter being able to suction around the cervix. These are little known issues, to the extent where patients experience hydronephrosis for multiple cycles before seeking medical attention.^25^

In 2020, a UK current affairs and debate programme named BBC’s Victoria Derbyshire highlighted that even with the addition of air holes, due to the suction effect of the rim, pulling the cup down to remove is the suspected cause for an unknown number of prolapses in the UK and around the world.^26^ There is no warning of this on the product’s safety label. Difficulty removing a menstrual cup increases when it is too large.

### Variation

Menstrual cups vary in size, shape, material, and firmness. Many brands offer two sizes (e.g. Fun Cup, Merula, Mooncup/MCUK), where another has 269 variations allowing users to select size, firmness, stem, and colour (Me Luna,^27^ December 2020). Menstrual cups are often categorized by their shape because it is one of the few ways women can judge a menstrual cup before it is purchased and used. This article recommends that menstrual cup shapes are generalized into v-shape, bell-shape, round-shape, and asymmetrical-shape. These categories are defined in Table 2.

**Table 2.** Menstrual cup shape category definitions.

Shape category	Example/Visual	Definition
V-shape	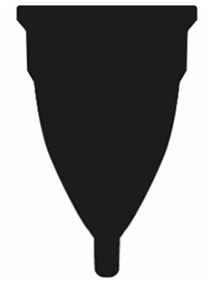	Menstrual cup vessel tapers in gradually from the rim to the stem, with the rim as the widest part of the menstrual cup. The vessel is longer than it is wide.
Bell-shape	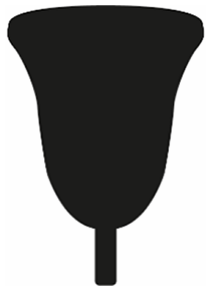	A rounder vessel that may flare up at the rim, with bell-shaped curves. The vessel is longer than it is wide.
Round-shape	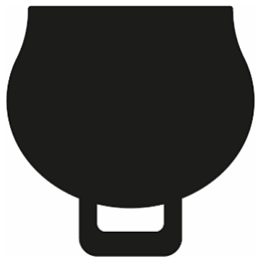	A more spherical-shaped vessel, the vessel is wider than it is long, with the widest point of the vessel being below the rim.
Asymmetrical-shape	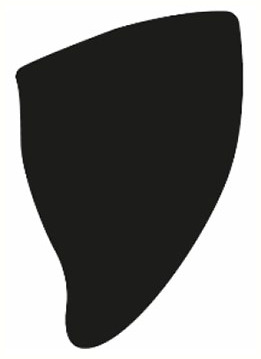	Any asymmetrical menstrual cup. These are designed to sit at a particular rotation and angle under the cervix, with the rim not necessarily being perpendicular to the axis of the vessel. The vessel is longer than it is wide.

Menstrual cups range in firmness, depending on their material and thickness. Some companies offer menstrual cups in varying firmness levels, including Lena (original and sensitive) and Me Luna (soft, classic, or sport). Manufacturers don’t provide metric information on firmness.

### Current study

There is no level of regulation and standardization of menstrual cups in terms of size, shape, volume, or firmness. With menstrual cup size and firmness having a direct effect on safety, usability, comfort, and leaks, it is important to identify acceptable physical and mechanical properties of menstrual cups, with categorization similar to the absorbency levels of tampons or sanitary pads. Standardization across menstrual cup types and brands would empower women to make the choices necessary for a successful menstrual cup experience and improve safety overall.

The aims of this menstrual cup comparison are to understand how menstrual cups across different brands compare in terms of general size, shape, material, volume, and firmness; to understand whether a consumer can estimate the volume of a menstrual cup judging by its general size or shape; and whether consumers can estimate a cup’s firmness based on a menstrual cup’s material or shape. It will also attempt to identify potential size and firmness categorization to improve safety and transparency to ultimately help women choose the most appropriate menstrual cup from the outset. This is the first study reporting an objective, quantitative comparison of 14 menstrual cups.

## Method

Quantitative, ex-vivo comparison of 14 menstrual cups was undertaken in laboratory setting at Nottingham Trent University, Nottingham, UK, in October 2020.

### Determination of menstrual cups to study

**Figure 2.**
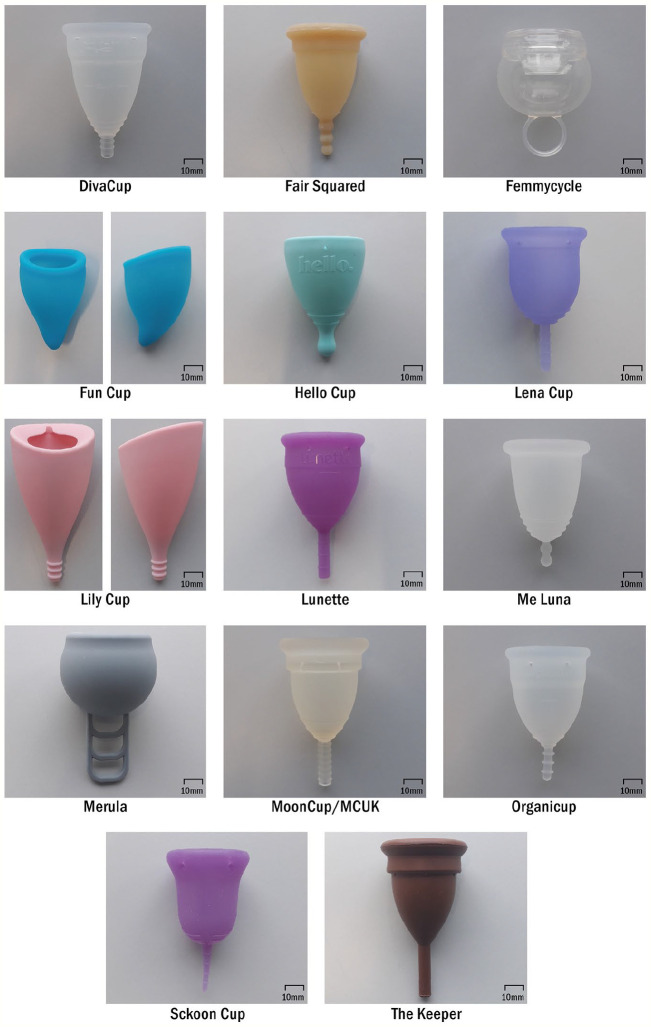
Menstrual cups compared in this study.

With no peer-reviewed or scientific information available, and being accessible to the general public, website MenstrualCupReviews^19^ was used as a reference to identify which menstrual cups to study. With almost 200 menstrual cups listed, it was the largest collection of objective menstrual cup information found online, found searching (‘menstrual cup’) AND rating OR review on Google. The following cups were chosen: DivaCup, Fair Squared, Femmycycle, Fun Cup, Hello Cup, Lena Cup, Lily Cup, Lunette, Me Luna, Merula, Mooncup/MCUK, Organicup, Sckoon Cup, and The Keeper (see Figure 2). A range of menstrual cups were chosen to span across the general shapes – v-shape, bell-shape, round-shape, and asymmetrical-shape – and across the three materials: silicone, TPE, and natural rubber. See Table 2 for shape category definitions, and Table 3 for the menstrual cup category matrix chosen for this study. A minimum of two menstrual cups in each category were chosen. Being the most prevalent material on the market, it was possible to study silicone menstrual cups from all four shape categories. Being the most prevalent shape on the market, it was possible to study v-shape menstrual cups in the three available materials. The highest-rated menstrual cups within each shape and material category were chosen, confirmed in April 2020. Therefore, within the round-shape category, for example, the menstrual cups are not as highly rated as other shapes.

**Table 3.** Menstrual cup shape and material categories.

Material	Shape			
	V-shape	Bell-shape	Round-shape	Asymmetrical-shape
Silicone	Lunette DivaCup Mooncup/MCUK Organicup	Sckoon Cup Lena Cup	Merula Femmycycle	Lily Cup Fun Cup
TPE	Me Luna Hello Cup			
Rubber	Fair Squared The Keeper			

TPE: thermoplastic elastomers.

Several cup manufacturers offer a variety of size options, which often includes two options: one smaller option for women less than the age of 30 years, with no history of pregnancy and childbirth, and a larger option for women above the age of 30 years, or with history of pregnancy and childbirth. However, some companies offer yet a smaller menstrual cup for teenagers or beginners (Me Luna, Organicup, Hello Cup), and some larger still (Me Luna). It was not possible to purchase every menstrual cup available. Therefore, one menstrual cup size was chosen from each manufacturer, advertised to women less than 30 years of age who have not given birth. This category was consistent across most menstrual cup manufacturers, even if the size category names were not consistent (i.e. size Small, size B), as seen in Table 4. There were two exceptions: from Hello Cup, ‘one size fits most’ size menstrual cup for women ‘under 35 years old and/or super sporty’ was chosen. From Me Luna, ‘Size M mainly used by women of all ages with normal muscles and medium flow’ was chosen.

**Table 4.** Menstrual cup comparison: models, dimensions, and volume.

Name	Size	Material	Shape	Total length (mm)	Vessel length (mm)	Stem length (mm)	Rim width (mm)	Maximum diameter (mm)	General size (mm^2^)	Volume to holes (mL)	Total volume (mL)
DivaCup	Model 1	Silicone	V-shape	66.00	56.00	10.00	42.50	42.50	2380.00	22.16	27.81
Fair Squared	Size M	Rubber	V-shape	65.20	48.20	17.00	40.80	40.80	1966.56	14.26	21.13
Femmycycle	Regular	Silicone	Round-shape	64.00	42.00	22.00	39.00	48.70	2045.40	. . .^a^	27.59^b^
Fun Cup	Size A	Silicone	Asymmetrical-shape	50.00	50.00	. . .^c^	37.90	41.00	2050.00	19.62	26.24
Hello Cup	Size S/M	TPE	V-shape	60.00	41.70	18.30	41.00	41.00	1709.70	21.00	25.44
Lena Cup	Small	Silicone	Bell-shape	71.00	47.50	23.50	41.00	41.00	1947.50	20.46	25.03
Lily Cup	Size A	Silicone	Asymmetrical-shape	78.00	68.00	10.00	. . .^d^	39.00	2652.00	. . .^a^	28.15
Lunette	Model 1	Silicone	V-shape	72.40	49.00	23.40	41.00	41.00	2009.00	18.57	25.06
Me Luna	Classic M^e^	TPE	V-shape	63.00	50.00	13.00	41.00	41.00	2050.00	18.01	23.92
Merula	. . .^f^	Silicone	Round-shape	73.00	40.00	33.00	40.00	46.00	1840.00	. . .^a^	38.14
Mooncup/MCUK	Size B	Silicone	V-shape	73.00	50.30	22.60	43.30	43.30	2177.99	17.18	24.71
OrganiCup	Size A	Silicone	V-shape	67.00	48.60	18.40	41.00	41.00	1992.60	20.02	28.06
SckoonCup	Size 1	Silicone	Bell-shape	70.50	45.20	25.30	40.00	40.00	1808.00	17.81	18.88
The Keeper	Size B	Rubber	V-shape	78.60	52.50	26.10	44.00	44.00	2310.00	13.03	24.22

*Note*. TPE: thermoplastic elastomers.

a no air holes; ^b^measured to flipped anti-spill lip; ^c^no stem; ^d^no rim; ^e^with stem; ^f^no specific name.

### Dimensions

Digital vernier callipers (RS Components) were used for all length measurements: total length, vessel length, stem length, rim width, and maximum diameter. Care was taken to not compress or distort the menstrual cups when measuring. It was also necessary to examine a menstrual cup’s general size because consumers are unlikely to know the actual volume of menstrual blood loss each month, and where sanitary pads and tampons have absorbency ratings to aid in correct product selection, menstrual cups do not. Consumers may therefore judge menstrual cup’s size visually. General size was quantified by a menstrual cup’s vessel length × vessel maximum diameter.

### Volume

To calculate menstrual cup volume, each menstrual cup was filled with water to the desired volume: volume to holes and total volume. Volume was calculated, with volume = mass/density, and 1 cm^3^ water weighing 1 g, measured using Kern PCB 1000 g × 0.01 g laboratory balance (Kern).

### Compressive strength

International Standards for contraceptive diaphragms include methods for regulating general quality and freedom from defects, minimal tensile strength, and compression and twisting resistance.^28^ Where these regulate the general safety, quality, and longevity of the product, none of the standards engage with comfort or usability. Menstrual cup mechanical properties could not simply be undertaken by comparing each product’s material’s shore hardness following testing standards in isolation to its overall design because these findings would not be translatable to real menstrual cup use. Product thickness, material, and overall shape heavily influence its firmness.

Compression testing was undertaken using an Instron Universal Testing System 3367 in compression mode, fitted with a 500 N load cell and using Bluehill 2 software control. The menstrual cup was placed between the centre of the plates and compressed just enough to hold in place at its maximum diameter. The product was compressed at a constant rate of 5 mm/min to 50% (±0.5%) of the menstrual cup’s maximum diameter. Each menstrual cup was compressed 5 times, rotating the menstrual cup approximately 30° after each test.

### Data analysis

A Shapiro-Wilk test examined whether continuous variables were normally distributed, and a means analysis was used to test homoscedasticity. Spearman’s rank-order correlation was undertaken. As sample sizes between the dependent variable groups were not equal, a nonparametric Levene’s test was used to test homogeneity of variances.^29^ A Kruskal–Wallis test was used when data were not normally distributed but displayed homogeneity of variances shown via a nonparametric Levene’s test, or when data was not homoscedastic. Quantitative data analysis was undertaken using IBM SPSS Statistics 26.0 (IBM Corp.). Data collection was undertaken in October 2020, and data analysis was undertaken in December 2020.

Ethical approval was not required for this study as it did not involve human participants, human tissues or data, or animals according to Nottingham Trent University Code of Practice for Research.

## Results

### Menstrual cup size, shape, material, volume, and compressive strength

Menstrual cup properties varied greatly across the differing brands, as shown in Tables 4 and 5. Menstrual cup total length ranged from 40.00 mm (Merula) to 78.60 mm (The Keeper; *M* = 67.98 mm, *SD* = 7.26 mm), and maximum diameter ranged from 39.00 mm (Lily Cup) to 48.70 mm (Femmycycle; *M* = 42.16 mm, *SD* = 2.49 mm).

**Table 5.** Menstrual cup comparison: compressive strength (firmness).

Name	Compressive load to compress menstrual cup 50% (±0.5%) maximum diameter/N					
	Compression cycle					*Mean* (*SD*)
	1	2	3	4	5	
DivaCup	6.12	5.58	5.68	5.06	7.67	6.02 (0.89)
Fair Squared	3.38	3.35	3.49	3.34	3.37	3.39 (0.05)
Femmycycle	12.01	11.83	11.84	11.60	11.77	11.81 (0.13)
Fun Cup	15.66	14.49	11.18	10.70	13.20	13.05 (1.89)
Hello Cup	13.28	14.38	14.06	13.12	14.74	13.92 (0.62)
Lena Cup	10.22	10.67	10.29	10.54	10.64	10.47 (0.18)
Lily Cup	5.89	5.92	5.37	5.16	5.13	5.49 (0.35)
Lunette	5.75	5.73	6.32	7.16	6.54	6.30 (0.53)
Me Luna	9.03	8.07	8.53	12.72	7.83	9.24 (1.79)
Merula	11.60	10.87	14.17	13.98	18.27	13.78 (2.59)
Mooncup/MCUK	6.26	6.39	5.92	5.46	5.82	5.97 (0.33)
Organicup	7.34	6.10	6.59	6.56	7.25	6.77 (0.46)
Sckoon Cup	8.60	8.55	8.83	8.69	8.49	8.63 (0.12)
The Keeper	8.20	9.18	9.04	8.94	8.83	8.84 (0.34)

Each menstrual cup underwent five separate compression cycles compressing each menstrual cup to 50% (±0.5%) maximum diameter, rotating the menstrual cup approximately 30° after each test; *SD*: standard deviation.

Volume to holes varied from 13.03 mL (The Keeper) to 22.16 mL (DivaCup; *M* = 18.37 mL, *SD* = 2.65 mL). Although volume to holes is a more meaningful value to rate a menstrual cup’s volume as it shows a true usable volume, three menstrual cups did not have air holes. Total volume is the only volume comparable across the full range of menstrual cups, and it ranged from 18.88 mL (Sckoon Cup) to 38.14 mL (Merula; *M* = 26.03 mL, *SD* = 4.21 mL).

**Figure 3.**
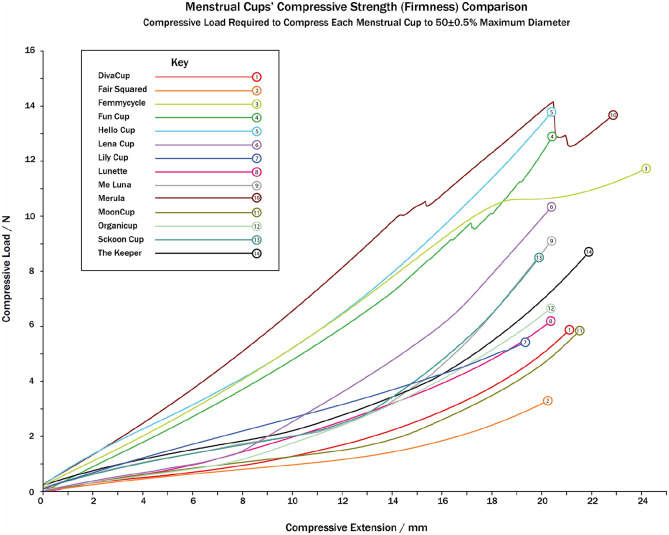
Menstrual cups’ compressive strength (firmness) comparison.

Menstrual cup firmness, measured by the compressive load required to compress the menstrual cup to 50% (±0.5%) maximum diameter measured in N (1 N = 1 kg m/s^2^), varied from 3.39 N (Fair Squared) to 13.92 N (Hello Cup; *M* = 8.83, *SD* = 3.25). Where Table 5 provides the total compressive load, menstrual cups behave differently when compressed: some menstrual cups compressed smoothly and gradually (DivaCup, Fair Squared, MoonCup/MCUK), whereas others compressed in an irregular fashion as seen in Figure 3 (Fun Cup, Merula). This article proposes that menstrual cup firmness is categorized by the following table to empower consumers to find a suitable menstrual cup (Table 6).

**Table 6.** Proposed menstrual cup firmness categories.

Category	Symbol	Compressive load required for 50% (±0.5%) diameter compression (N)	Brand examples
Very soft	⚫	x ⩽ 5.2	Fair Squared
Soft	⚫⚫	5.2 < x ⩽ 7.4	DivaCup, Lily Cup, Lunette, Mooncup/MCUK, Organicup
Medium	⚫⚫⚫	7.4 < x ⩽ 9.6	Me Luna, Sckoon Cup, The Keeper
Firm	⚫⚫⚫⚫	9.6 < x ⩽ 11.8	Lena Cup
Very firm	⚫⚫⚫⚫⚫	11.8 < x ⩽ 14.0	Femmycycle, Fun Cup, Hello Cup, Merula

### General size and volume

A Spearman’s correlation test showed there was no statistically significant correlation between menstrual cup’s general size and its volume, *r*s(14) = .18, *p* = .53.

### Shape and volume

A Kruskal–Wallis test showed that distribution of volume was not significantly different across menstrual cup shape categories, χ^2^(3) = 6.00, *p* = .11.

### Material and compressive strength

A Kruskal–Wallis test showed that distribution of firmness was not significantly different across menstrual cup material categories, χ^2^(2) = 2.88, *p* = .24.

### Shape and compressive strength

A Kruskal–Wallis test showed that distribution of firmness was not significantly different across menstrual cup shape categories, χ^2^(3) = 3.17, *p* = .37.

## Discussion

Choosing the 14 most popular and highly rated menstrual cups according to MenstrualCupReviews,^19^ this study was a starting point to explore how menstrual cups across different brands compare in terms of design, material, and mechanical and physical properties.

In this comparison of 14 menstrual cups, dimensions varied greatly, with total length ranging from 40.0 mm (Merula) to 78.6 mm (The Keeper), and maximum diameter ranging from 39.0 mm (Lily Cup) to 48.7 mm (Femmycycle). Total volume ranged from 18.88 mL (Sckoon Cup) to 38.14 mL (Merula), meaning users will be required to empty the Sckoon Cup around twice as often as the Merula with the same rate of menstrual blood loss. All the menstrual cups tested here were marketed to women less than the age of 30 years having never given birth, and as the range in shape and size in Figure 1 shows, menstrual cup variations across the brands could be confusing. General menstrual cup size does not relate to its volume, so size can be misleading. There should be some guidance or structure for comparing menstrual cup size, which was an aim for this article. However, as there is no correlation between general size and volume, it is unclear whether it would be more suitable to categorize menstrual cups by volume, allowing consumers to judge by their menstrual blood loss. This might result in a suitable volume not being a suitable shape for the consumer: too wide or too long. Conversely, categorizing menstrual cups by size, allowing consumers to choose based on their vaginal length or cervix height, for example, will not reflect its volume. Similarly, consumers choosing the visually ‘largest’ menstrual cup thinking that it will meet their heavy menstrual flow may be disappointed. International Standards for contraceptive diaphragms ISO 2014 contraceptive regulate dimensions of vaginally worn contraceptive devices, specifying that size must sit within certain limits, from 55 mm to 100 mm,^28^ but this is not applicable to menstrual cups. If menstrual cup size was categorized in a similar way, and this categorization standardized across brands, it would provide transparency for women to make informed decisions. Size and volume categorization remain an unanswered question in this article.

Firmness measured by the compressive load required to compress the menstrual cup to 50% (±0.5%) maximum diameter varied from 3.39 N (Fair Squared) to 13.92 N (Hello Cup). This means one menstrual cup marketed to a nonparous 29-year-old will be too soft and not open inside the vagina, causing leaks, and another would be too firm, may feel uncomfortable, and could potentially cause injury. Material or shape of menstrual cup was not correlated to its firmness, meaning women would not be able to estimate which would be most suitable based on these. On the product packaging, Sckoon Cup advertises its product as ‘the softest and most advanced menstrual cup’. However, Sckoon Cup was found to sit within the ‘medium’ firmness category in this study. This work recommends manufacturers clearly label products with firmness categories, proposed in Table 6. A firmness comparison chart and clarity on packaging would prevent any misconceptions. This work proposes the first recommended categorization of menstrual cups into firmness categories: ranging from, ‘very soft’, ‘soft’, ‘medium’, ‘firm’, and ‘very firm’. In the same way that tampons are categorized in terms of absorbency for ease of use and comfort, as well as reducing risk of toxic shock syndrome,^30^ these categories can improve consumers’ comfort and safety. If more responsibility lies with manufacturers to display the firmness of the menstrual cups, and practice shifts to educate healthcare practitioners to be able to support women in finding a suitable menstrual cup, women will find greater success and improved safety in using one. There will also be better understanding of the prevalence of injuries such as prolapse as a result of using a menstrual cup, as well as accurate reportings of adverse incidents if healthcare professionals are more engaged in the process.

With more research, a user’s age, history of childbirth, body mass index, and level of fitness could indicate which firmness category would be most suitable, removing the element of trial and error, and improving menstrual cup user experience.

### Strengths

This study was undertaken in a laboratory setting, offering a high level of control over the environment and variables measured. Along with being replicable, just as the Food and Drug Administration (FDA) compare tampons in a laboratory setting when establishing their absorbency levels,^30^ these methods can be undertaken in comparable settings for other researchers and menstrual cup manufacturers to accurately assess menstrual cup properties. This study is the first step to demystifying menstrual cup size, shapes, material, and firmness, with the aims for manufacturers to clearly present this information. This will improve comfort and safety, as the knowledge will help women know which menstrual cup might be well suited. In particular, the quantification of menstrual cup firmness allows women to clearly compare menstrual cups to make informed decisions.

### Limitations and future study

Only 14 menstrual cup brands were chosen for this first comparison. Exploring inter-brand menstrual cup variations, particularly varying firmness categories, is missing from this study. By only picking the most popular and highly rated menstrual cup brands, this study is missing the lesser-known or newer brands, and importantly the very inexpensive menstrual cups that have been criticized for being potentially dangerous.^31^

Size and volume categorization remain an unanswered question in this study, and it must be explored in future studies to identify how menstrual cups can be regulated properly. It is recommended future studies categorize all brands in terms of shape, material, size, total volume, firmness, and so on in a single table for easier decision-making by consumers. This is the first objective, quantitative comparison of 14 menstrual cups. The understanding of menstrual cup’s physical and mechanical properties is a first step to later identify whether certain menstrual cups hold inherently more risk of injury than others.

Compression testing was limited. Testing was undertaken at five points at room temperature, rotating the menstrual cup approximately 30° after each test. It does not represent the dynamic, three-dimensional compression of the vaginal canal. Where this study is an important initial comparison, future analysis of the mechanical properties of menstrual cups could be undertaken in more a realistic, dynamic setting. The FDA ‘Syngyna testing’ method could be utilized:^30^ a three-dimensional vagina model currently used to hold tampons in place as ‘syngyna’ fluid is used to measure tampon absorbency. This can also be undertaken at 37 °C.

Van Eijk et al.^2^ identified that menstrual cup brands ranged in price from US$0.72 to US$46.72. Menstrual cup cost was not included in this analysis. Product cost will ultimately have an influence on a consumer’s decision. Future analysis should measure whether product cost is associated with either menstrual cup uptake or factors such as material quality, product longevity, or comfort.

It was found that increased support and education in reproductive anatomy might improve menstrual cup use willingness and success.^14,32^ This increased support and education could come from medical professionals, but future work should also explore how girls and women without access to medical professionals, including those living in refugee camps and low-income settings, can find a suitable menstrual cup. Future study is needed to show how an improved knowledge on reproductive anatomy could also help women choose which menstrual cup might be more suitable. It would then be important to examine which factors are most significant in terms of safety and acceptability. Future study is needed to explore what women themselves are looking for when choosing a menstrual cup and how they would prefer to be supported in this experience.

## Conclusion

This work is the first objective comparison of 14 of the most popular and highly rated menstrual cups of differing shapes and materials, comparing DivaCup, Fair Squared, Femmycycle, Fun Cup, Hello Cup, Lena Cup, Lily Cup, Lunette, Me Luna, Merula, Mooncup/MCUK, Organicup, Sckoon Cup, and The Keeper, which vary greatly. There is no correlation between a menstrual cup’s size, shape, and volume, or a menstrual cup’s material, shape, and firmness. Women guess which shape or material might suit them, and if incorrect may experience discomfort, leaks, and increase the risk of injury. More research is clearly needed to further empower women to choose the correct menstrual cup, and improve their regulation from the FDA, the Medicines and Healthcare products Regulatory Agency, and similar regulatory authorities worldwide. Women need more support and guidance from healthcare providers when choosing a menstrual cup to make better decisions in their reproductive life.
